# Circulating Soluble EPCR Levels Are Reduced in Patients with Ischemic Peripheral Artery Disease and Associated with Markers of Endothelial and Vascular Function

**DOI:** 10.3390/biomedicines11092459

**Published:** 2023-09-04

**Authors:** Janina Krug, Magdalena L. Bochenek, Rajinikanth Gogiraju, Dagmar Laubert-Reh, Karl J. Lackner, Thomas Münzel, Philipp S. Wild, Christine Espinola-Klein, Katrin Schäfer

**Affiliations:** 1 Department of Cardiology, Cardiology I, University Medical Center Mainz, 55131 Mainz, Germanymagdalena.bochenek@unimedizin-mainz.de (M.L.B.); rajinikanth.gogiraju@unimedizin-mainz.de (R.G.); dagmar.laubert-reh@unimedizin-mainz.de (D.L.-R.); tmuenzel@uni-mainz.de (T.M.); philipp.wild@unimedizin-mainz.de (P.S.W.); 2 Center for Thrombosis and Hemostasis, University Medical Center Mainz, 55131 Mainz, Germany; 3 Institute of Clinical Chemistry and Laboratory Medicine, University Medical Center Mainz, 55131 Mainz, Germany; karl.lackner@unimedizin-mainz.de; 4 Department of Cardiology, Cardiology III, University Medical Center Mainz, 55131 Mainz, Germany; espinola@uni-mainz.de

**Keywords:** angiogenesis, coagulation, endothelium, ischemia, peripheral artery disease

## Abstract

(1) Background: Endothelial dysfunction initiates cardiovascular pathologies, including peripheral artery disease (PAD). The pathophysiology of impaired new vessel formation in the presence of angiogenic stimuli, such as ischemia and inflammation, is unknown. We have recently shown in mice that reduced endothelial protein C receptor (EPCR) expression results in defective angiogenesis following experimental hindlimb ischemia. (2) Purpose: To determine soluble (s)EPCR levels in the plasma of patients with PAD and to compare them with the protein C activity and biomarkers of endothelial function, inflammation, and angiogenesis. (3) Methods and Results: Clinical tests of vascular function and immunoassays of plasma from patients with PAD stage II were compared to age- and sex-matched individuals with and without cardiovascular risk factors or PAD stage III/IV patients. sEPCR levels were significantly lower in PAD stage II patients compared to subjects with risk factors, but no PAD, and further decreased in PAD stage III/IV patients. Plasma protein C activity or levels of ADAM17, a mediator of EPCR shedding, did not differ. Significant associations between sEPCR and the ankle-brachial index (*p* = 0.0359), age (*p* = 0.0488), body mass index (*p* = 0.0110), and plasma sE-selectin levels (*p* = 0.0327) were observed. High-sensitive CRP levels and white blood cell counts were significantly elevated in PAD patients and associated with serum glucose levels, but not sEPCR. In contrast, plasma TNFα or IL1β levels did not differ. Circulating levels of VEGF were significantly elevated in PAD stage II patients (*p* = 0.0198), but not associated with molecular (sE-selectin) or functional (ankle-brachial index) markers of vascular health. (4) Conclusions: Our findings suggest that circulating sEPCR levels may be useful as biomarkers of endothelial dysfunction, including angiogenesis, in persons older than 35 years and that progressive loss of endothelial protein C receptors might be involved in the development and progression of PAD.

## 1. Introduction

The anticoagulant properties of the endothelium are mediated, among others, by the endothelial protein C receptor (EPCR), that binds and activates protein C, a circulating anticoagulant serine protease [1]. EPCR and its co-receptor thrombomodulin are abundantly expressed on healthy, ‘functional’ endothelial cells lining capillaries, arteries, and veins. Whereas circulating levels of thrombomodulin in healthy individuals reflect the quantity of the receptor expressed on the endothelial cell surface, an increased proteolytic activity associated with endothelial dysfunction enhances its shedding from endothelial cells [2]. Based on these and other findings [3,4,5], soluble thrombomodulin levels have been suggested as a marker of endothelial dysfunction and were found to be significantly elevated in patients with peripheral occlusive arterial or ischemic heart disease [6] or in patients with myocardial infarction suffering a pre-defined endpoint during follow-up [7]. Inflammatory mediators, such as tumor necrosis factor-alpha (TNFα), interleukin-1 beta (IL1β), or thrombin have been shown to increase EPCR shedding from cultivated endothelial cells [8,9]. However, and in contrast to thrombomodulin, little is known about the impact of endothelial dysfunction on the expression of EPCR on endothelial cells in the context of peripheral artery disease (PAD). A previous study found significantly higher baseline soluble EPCR (sEPCR) levels in patients with acute, ST-elevation myocardial infarction (STEMI) compared to patients with stable angina pectoris [10]. Importantly, first data in mice suggest that inhibition of EPCR shedding, for example by the glucagon-like peptide-1 receptor agonist exendin-4 [11], may improve myocardial infarct size in pigs [12] or in patients with STEMI [13]. We have recently shown that genetic deletion of EPCR in the murine endothelium impairs revascularization following experimental induction of hindlimb ischemia [14]. This clinical pilot study aimed to determine soluble EPCR levels and factors involved in EPCR shedding in patients with PAD and their association with other, established biomarkers of endothelial or vascular function.

## 2. Materials and Methods

### 2.1. Study Population

Between October 2019 and March 2021, 20 consecutive patients (n = 10 male, n = 10 female) diagnosed with peripheral artery disease (PAD) were included in the study. Inclusion criteria were the presence of symptomatic intermittent claudication, classified based on clinical criteria, an ankle-brachial index of less than 0.9, and a closing pressure drop during treadmill ergometry (study group ‘PAD stage II’), according to Fontaine and extended by Rutherford [15,16]. Exclusion criteria were: age under 18 years, lack of informed consent, malignancy with a life expectancy limited to less than 12 months, immunosuppression, febrile infectious disease within the last 4 weeks, impaired renal function (estimated creatinine clearance < 30 mL/min), or pregnancy. At a later stage, 20 patients with critical limb ischemia, defined as pain at rest or visible ulcerations or necrosis (Fontaine grade III and IV; study group ‘PAD stage III/IV’) were prospectively included for comparison, based on the age and sex of the PAD stage II group. All PAD patients were recruited via the Department of Cardiology, Cardiology III (Angiology) at the University Medical Center Mainz, Germany.

Control groups consisted of age- and sex-matched individuals without a diagnosis of PAD, either with a similar cardiovascular risk profile (study group ‘healthy, age-matched with risk factors’; n = 20) or without cardiovascular risk factors (study group ‘healthy, age-matched’; n = 20). These individuals were recruited via the Gutenberg Health Study (GHS), a population-based, prospective, observational, single-center cohort study in the Rhine-Main region of western mid-Germany [17,18]. Cardiovascular risk factors were defined as follows: diabetes mellitus type 2 was defined as fasting glucose of 126 mg/dL or higher or antidiabetic medication/insulin therapy, according to [19]; arterial hypertension was defined as blood pressure of 140/90 mmHg and greater or antihypertensive medication, according to [19]; metabolic syndrome was defined as BMI > 30 kg/m^2^ plus 2 out of the following 4 factors: triglyceride levels 150 mg/dL or greater, HDL cholesterol <50 mg/dL in women and <40 mg/dL in men, blood pressure 130/85 mmHg or higher, fasting serum glucose 100 mg/dL or greater, according to the consensus statement of the International Diabetes Federation [20] and the National Heart, Lung, and Blood Institute/American Heart Association [21].

In addition, 20 sex-matched volunteers aged 35 years or younger with no known cardiovascular risk factors or disease were included in the study (study group ‘healthy < 35 years’). It should be noted that one patient in the PAD stage II group (a 62-year-old female) as well as their age- and sex-matched pairs were excluded from the study, the rationale is explained in the Section 3. Consequently, the final number of patients or persons changed to n = 19 per group.

### 2.2. Perfusion Tests

A non-invasive functional angiological basic diagnostic work-up was carried out as part of the routine examination in all patients with PAD. This included the determination of the ankle-brachial index, continuous-wave Doppler sonography, and electronic oscillography, as described earlier [22,23] in all PAD patients. In addition, treadmill ergometry was performed at 3.2 km/h and a 12% incline in patients with PAD stage II. These examinations were not indicated (and therefore not performed) for individuals of both control groups and individuals 35 years of age or younger.

### 2.3. Blood Sampling, Serum, and Plasma Preparation

Peripheral venous blood was taken from all study participants during a routine clinic visit or at the time of hospitalization. For the preparation of plasma and its subsequent analysis, blood was collected in plastic tubes containing either lithium heparin (Sarstedt; catalog number 135469; for sEPCR, ADAM17, sE-selectin, TNFα, and CRP measurements) or EDTA (Sarstedt; catalog number 125312; for IL1β and VEGF measurements), or sodium citrate (Sarstedt; catalog number 125311; for protein C activity determination) as anticoagulants, as recommended by the manufacturer. Serum was prepared by allowing whole blood to clot for 2 h at room temperature. Plasma or serum were obtained by centrifugation at 3000× *g* for 10 min at room temperature. The supernatant was transferred to clean tubes, immediately divided into aliquots (500 µL), and frozen at −80 °C pending further analysis.

### 2.4. Routine Laboratory Methods

Whole blood cell counts, serum glucose and lipid levels (total cholesterol, high-density lipoprotein [HDL] and low-density lipoprotein [LDL]-cholesterol, triglycerides), C-reactive protein, and protein C activity were measured in all study participants using automated clinical chemistry analyzers. C-reactive protein (CRP) was determined by a highly sensitive, latex particle-enhanced immunoassay (Roche Diagnostics, Basel, Switzerland). These analyses were performed at the Institute for Clinical Chemistry, University Medical Center Mainz, Germany.

### 2.5. Enzyme-Linked Immunoassays

Commercially available enzyme-linked immunoassays (ELISAs) were used to determine the plasma levels of soluble EPCR (MyBioSource; MBS703733; sensitivity: 1.95 ng/mL), soluble E-selectin (R&D Systems; DSLE00; sensitivity: 0.027 pg/mL), IL1β (R&D Systems; DLB50; sensitivity: 1 pg/mL), TGFβ1 (R&D Systems; DB100B; minimal detectable dose: 1.7–15.4 pg/mL), TNFα (R&D Systems; DTA00D; sensitivity: 6.23 pg/mL), Tumor necrosis factor-alpha-converting enzyme (TACE/ADAM17; Thermo Scientific; EDADAM17; sensitivity: 70 pg/mL), soluble Tie2 (R&D Systems; DTE200; minimal detectable dose: 0.001–0.066 ng/mL), and VEGF (R&D Systems; DVE00; minimal detectable dose: 5–9 pg/mL). Plasma was used undiluted (sEPCR, TNFα, VEGF) or diluted 1:2 (ADAM17), 1:10 (sE-selectin), or 1:20 (TGFβ1), respectively. Latent TGFβ1 was activated, according to the manufacturer’s instructions, immediately before the analysis. To eliminate interassay variability, plasma aliquots were thawed and assayed on the same day. All analyses were performed in duplicate. Results were calculated from a standard curve created using computer software capable of generating two-dimensional graphs using logarithmic scales (log–log), or four-parameter logistic (4-PL) curve fit for CRP values.

### 2.6. Statistical Analysis

All statistical analyses were done using GraphPad Prism software (version 9.5.0 for Windows, GraphPad Software, Boston, MA, USA, www.graphpad.com, accessed on 1 July 2023). Quantitative data are reported as mean ± standard deviation (SD) and are shown as violin blots. Normal distribution was examined using the Shapiro–Wilk normality test. For the comparison of two groups and normal distribution, Student’s *t*-test was used. More than two groups were compared with One-Way Analysis of Variance (ANOVA) tests, followed by Sidak’s or Tukey’s multiple comparison tests, as recommended by the statistical program. Non-parametric tests (Mann–Whitney for the comparison of two groups and Kruskal–Wallis, followed by Dunn’s multiple comparisons test for more than two groups) were used if normal distribution was not present. Outlier analysis was performed for the primary endpoint of the study (sEPCR levels in plasma) using the ROUT method, setting Q to 1%. Simple linear regression was used to estimate the relationship between two quantitative data sets, using sEPCR as the independent variable. Categorical values are reported as a number (n) and percentage (%) and are compared using Chi-Square test. Statistical differences were assumed if *p* reached a value less than 0.05.

## 3. Results

Previous work from our and other laboratories in mice and cells suggested a role for EPCR on endothelial cells for new vessel formation during ischemia [14,24,25], and that EPCR expression is downregulated in ischemic muscle tissue from patients with peripheral artery disease [14]. To explore the usefulness of soluble EPCR shed from endothelial cells into the circulating blood as a potential biomarker of endothelial function and angiogenic potential, patients with PAD stage II were prospectively recruited and compared to age- and sex-matched healthy individuals, with or without cardiovascular risk factors, or to PAD stage III/IV patients. The clinical characteristics of the study collective are shown in Table 1 and routine laboratory parameters, including whole blood cell counts, and lipid and glucose levels are shown in Table 2. PAD patients and age- and sex-matched persons (with and without risk factors) exhibited a significantly higher mean BMI compared to individuals 35 years of age or younger (Table 1). Patients with PAD stage III/IV showed increased levels of biomarkers of inflammation, that is, elevated white blood cell counts or C-reactive protein levels, compared to all other groups. Conversely, red blood cell counts were significantly lower in PAD stage III/IV patients compared to age-matched individuals without risk factors and healthy persons < 35 years. Serum glucose levels were also significantly elevated, in line with chronic inflammation fueling insulin resistance [26]. Total and LDL cholesterol levels were highest in age-matched individuals without PAD diagnosis, in agreement with the higher percentage of patients taking cholesterol-lowering medication, whereas HDL cholesterol levels did not significantly differ between the groups (Table 2).

### 3.1. Soluble EPCR Levels in Plasma Are Reduced in Patients with Peripheral Artery Disease

**Figure 1:**
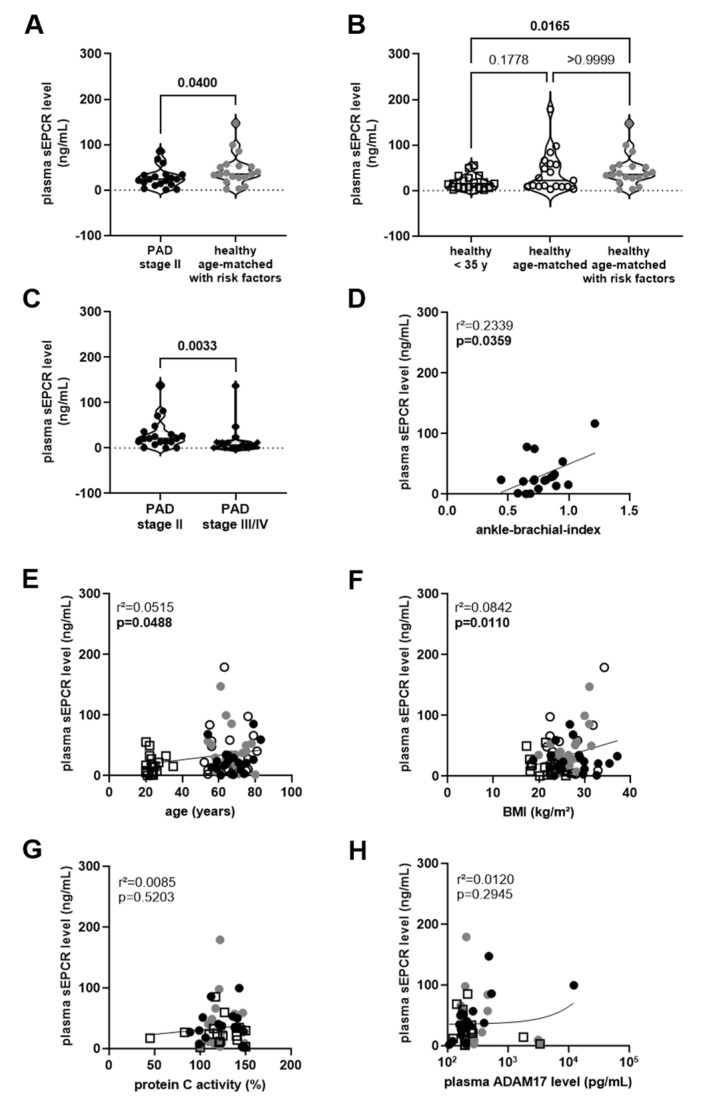
**Soluble EPCR level in plasma: association with PAD severity and cardiovascular risk factors.** (**A**), Levels of soluble endothelial protein receptor C (sEPCR) were determined in the plasma of patients with peripheral artery disease (PAD) stage II (n = 19; ●) and age- and sex-matched persons with cardiovascular risk factors and no PAD diagnosis (n = 19; 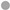). The results of the statistical analysis (using Mann–Whitney test) are shown within the graph. (**B**), Plasma sEPCR levels in age- and sex-matched healthy persons with (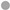) and without (◯) cardiovascular risk factors and in sex-matched individuals 35 years of age or younger (□). The results of the statistical analysis (using Kruskall–Wallis, Dunn’s multiple comparisons test) are shown within the graph. (**C**), Comparison of plasma sEPCR levels in patients with PAD stage II (●) and stage III/IV (♦), examined within the same experiments. The results of the statistical analysis (using Mann–Whitney test) are shown within the graph. Simple linear regression analysis of the association of plasma sEPCR levels with the ankle-brachial index (**D**), age (**E**), body mass index (BMI; (**F**)), and plasma protein C activity (**G**) or ADAM17 levels (**H**) was performed. The r-squared and the *p*-value for each analysis are shown within the graphs. Note that the ankle-brachial index was available only in PAD stage II patients (●), age and BMI also in age- and sex-matched healthy persons with (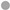) and without (◯) cardiovascular risk factors and in sex-matched individuals 35 years of age or younger (□). Significant differences are highlighted in bold.

ELISA analysis revealed that sEPCR protein levels circulating in the plasma of patients with PAD stage II were significantly lower than those in age- and sex-matched individuals with cardiovascular risk factors and no PAD diagnosis (*p* = 0.0400; Figure 1A). Because one of the measured sEPCR values in a PAD stage II patient (a 62-year-old female; 152.7 ng/mL; median value in all other patients: 23.5 ng/mL) was identified as an outlier by the statistical software program, this and all other data from this patient as well as their age- and sex-matched pairs were excluded from all further analysis. A comparison of sEPCR levels in healthy individuals revealed that sEPCR levels were significantly higher in age-matched persons with risk factors compared to healthy individuals 35 years of age and younger (*p* = 0.0165). In contrast, they did not differ from those in age-matched individuals without risk factors (Figure 1B).

To test the hypothesis that progressive endothelial dysfunction and loss of endothelial EPCR expression may underlie the observed reduction in sEPCR levels, age- and sex-matched patients with PAD stage III/IV were prospectively recruited. ELISA analysis showed that sEPCR plasma levels further decreased in patients with a more advanced PAD stage, and sEPCR plasma levels were significantly lower in PAD stage III/IV patients compared to plasma of PAD stage II patients, re-examined on the same plate (*p* = 0.0033; Figure 1C). Importantly, a significant association between plasma sEPCR levels and the ankle-brachial index, measured only in PAD stage II patients and contraindicated in PAD stage III/IV patients, was observed (r^2^ = 0.2339; *p* = 0.0359; Figure 1D), and plasma sEPCR were also significantly associated with the age (r^2^ = 0.0515; *p* = 0.0488; Figure 1E) and the BMI (r^2^ = 0.0842; *p* = 0.110; Figure 1F), but not with the protein C activity (r^2^ = 0.0085; *p* = 0.5203; Figure 1G) or the ADAM17 levels (r^2^ = 0.0120; *p* = 0.2945; Figure 1H) in plasma.

### 3.2. Elevated Biomarkers of Endothelial Dysfunction in Patients with Peripheral Artery Disease

**Figure 2:**
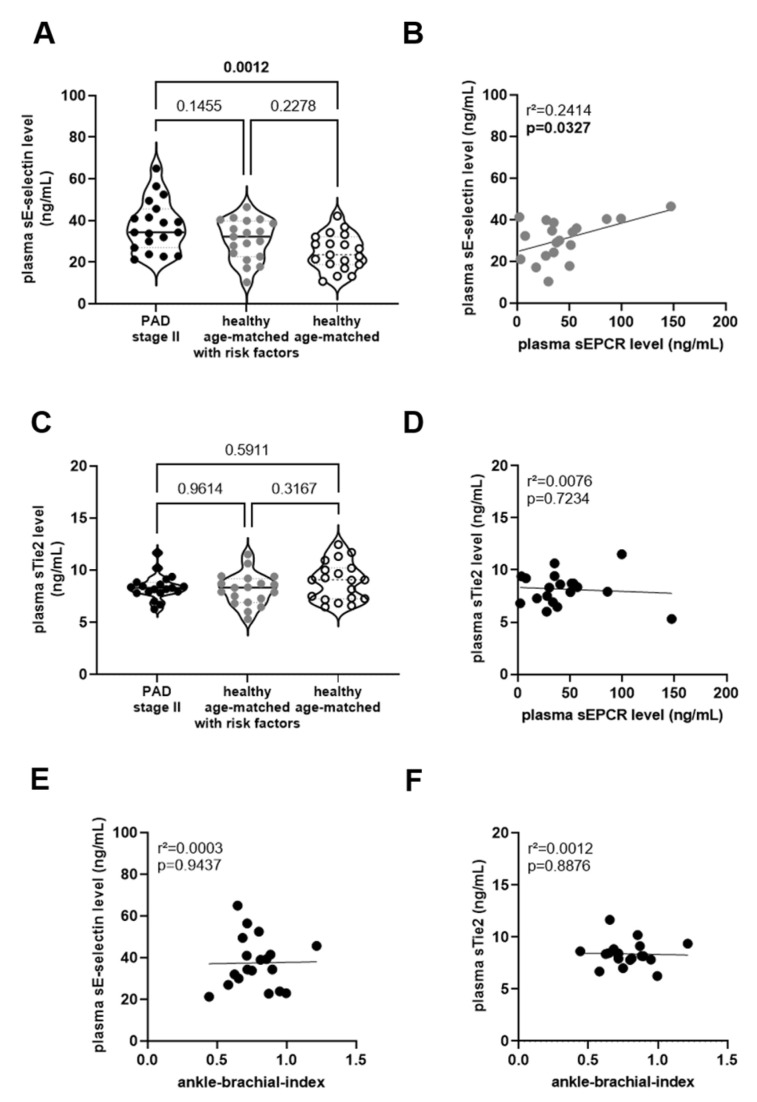
**Plasma markers of endothelial dysfunction.** Plasma levels of sE-selectin (**A**) and sTie2 (**C**) were measured in PAD stage II patients (●) and in age- and sex-matched healthy persons with (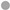) and without (◯) cardiovascular risk factors. The results of the statistical analysis (using One-Way-ANOVA, Sidak’s multiple comparisons test) are shown within the graph. Simple linear regression analysis of the association of plasma sEPCR and sE-selectin levels in age-matched person with risk factors (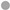; (**B**)) or with sTie2 levels in PAD stage II patients (●; (**D**)). Simple linear regression analysis of the association of plasma sE-selectin (**E**) or sTie2 (**F**) levels with the ankle-brachial index, determined in PAD stage II patients (●). The r-squared and the *p*-values are shown within the graphs. Significant differences are highlighted in bold.

Next, we tested the performance of established biomarkers of endothelial dysfunction in our study collective. Plasma levels of soluble E-selectin were significantly higher in PAD stage II patients compared to healthy age- and sex-matched individuals without PAD diagnosis and no cardiovascular risk factors (*p* = 0.0012), but did not differ compared to those with cardiovascular risk factors (Figure 2A). In line, a significant association between plasma sE-selectin and sEPCR levels was present in healthy, age-matched individuals with risk factors (r^2^ = 0.2414; *p* = 0.0327; Figure 2B), but absent in those without risk factors (r^2^ = 0.0904; *p* = 0.2109; not shown) or in PAD patients stage II (r^2^ = 0.0945; *p* = 0.2003; not shown). Plasma levels of Tie2, a receptor tyrosine kinase expressed on endothelial cells with barrier protective [27] and anti-inflammatory effects [28], did not significantly differ between PAD stage II patients and age-matched individuals without a PAD diagnosis (Figure 2C). Plasma levels of sTie2 were significantly associated with age (*p* = 0.0042; not shown), but not with sEPCR levels in patients with PAD stage II (Figure 2D). Notably, and in contrast to sEPCR, no association between plasma levels of sE-selectin (r^2^ = 0.0003; *p* = 0.9437; Figure 2E) or sTie2 (r^2^ = 0.0012; *p* = 0.8876; Figure 2F) and the ankle-brachial index was observed.

### 3.3. Increased Inflammatory Biomarkers in Patients with Cardiovascular Risk Factors

**Figure 3:**
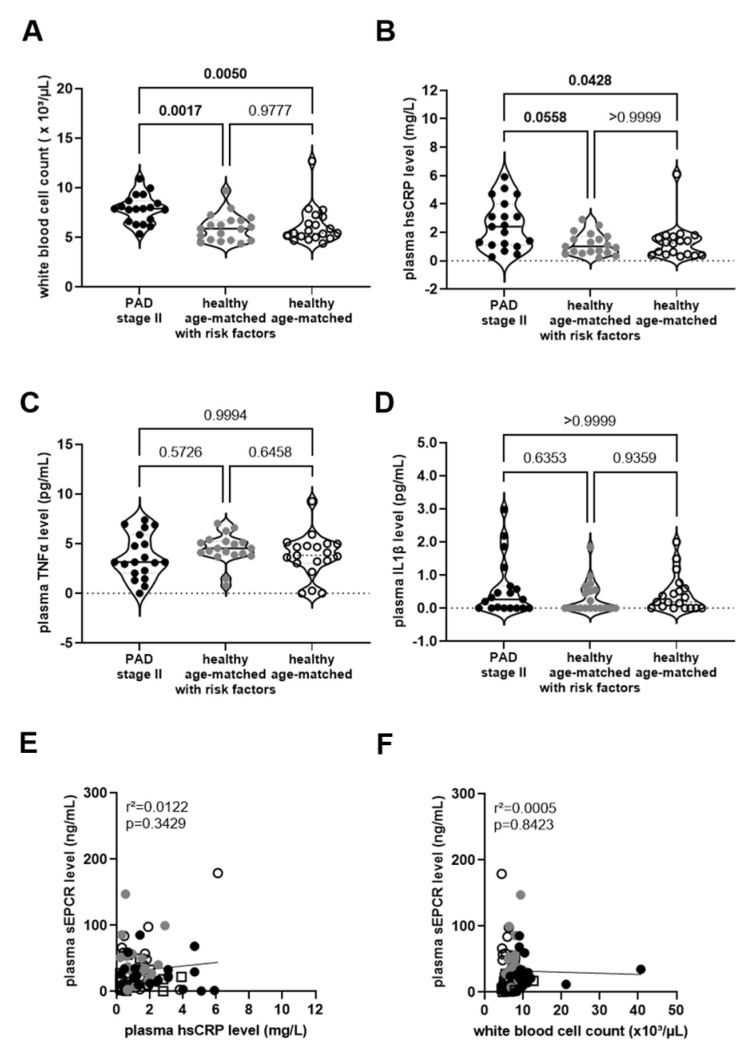
**Biomarkers of inflammation and their association with sEPCR.** (**A**), White blood cell (WBC) counts were determined in whole blood of PAD stage II patients (**●**) and in age- and sex-matched healthy persons with (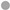) and without (**◯**) cardiovascular risk factors. Plasma from these groups was also examined for the concentrations of high-sensitive C-reactive protein (hsCRP; (**B**)), tumor necrosis factor-alpha (TNFα; (**C**)), or interleukin-1beta (IL1β; (**D**)). The results of the statistical analysis ((**A**,**C**): One-Way-ANOVA, Sidak’s multiple comparisons test; (**B**,**D**): Kruskall–Wallis, Dunn’s multiple comparisons test) are shown within the graph. Simple linear regression analysis of the association of sEPCR and hsCRP levels in plasma (**E**) or with the white blood cell count in whole blood (**F**). In sex-matched individuals 35 years of age or younger (□). The r-squared and the *p*-values are shown within the graphs. Significant differences are highlighted in bold.

EPCR has been associated with anti-inflammatory activities [29,30], whereas proinflammatory cytokines have been shown to promote endothelial EPCR shedding [8,9]. In this regard, the highest levels of biomarkers of inflammation, that is white blood cell counts (Figure 3A) and hsCRP (Figure 3B), were observed in PAD stage II patients compared to age- and sex-matched individuals, both with (*p* = 0.0017 and *p* = 0.0558, respectively) and without (*p* = 0.0050 and *p* = 0.0428, respectively) risk factors and no PAD diagnosis. Conversely, plasma TNFα levels did not significantly differ between the study groups (Figure 3C). Plasma levels of IL-1β were low or undetectable in many study participants and also did not differ between the study groups (Figure 3D). No significant associations were observed between plasma sEPCR and hsCRP levels (Figure 3E) or plasma sEPCR levels and white blood cell counts (Figure 3F). Conversely, WBC counts (*p* = 0.0005; not shown) and hsCRP levels (*p* = 0.0631; not shown) were associated with serum glucose levels, in line with metabolic inflammation.

### 3.4. Biomarkers of Angiogenesis and Anti-Angiogenesis in Peripheral Artery Disease Are Elevated, but Not Associated with Biomarkers of Endothelial or Vascular Function

**Figure 4:**
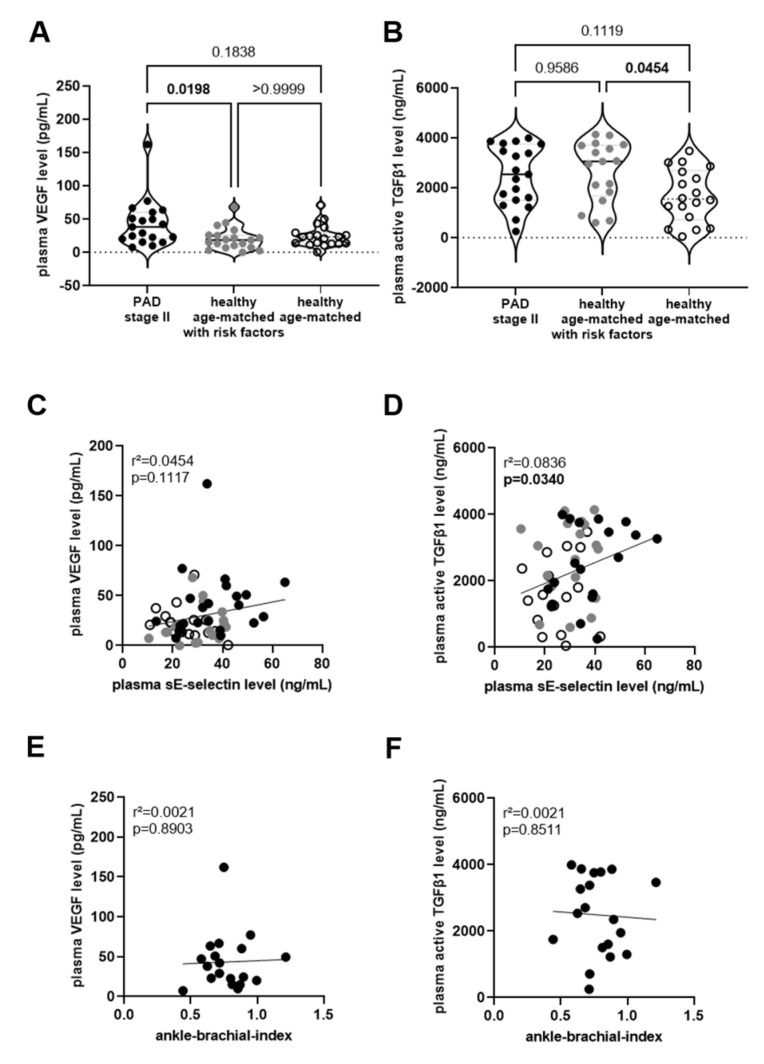
**Plasma levels of angiogenic growth factors and relation with endothelial function.** Plasma levels of vascular endothelial growth factor (VEGF; **A**) and active transforming growth factor-beta (TGFβ1; (**B**)) were determined in PAD stage II patients (●) and in age- and sex-matched healthy persons, with (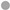) and without (◯) cardiovascular risk factors. The results of the statistical analysis (A: Kruskall–Wallis, Dunn’s multiple comparisons test; B: One-Way ANOVA, Sidak’s multiple comparisons test) are shown within the graph. Simple linear regression analysis to determine the association of VEGF (**C**) or active TGFβ1 (**D**) with sE-selectin levels in plasma of PAD stage II patients (●) and in age- and sex-matched healthy persons, with (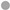) and without (◯) cardiovascular risk factors, and of plasma VEGF (**E**) or active TGFβ1 (**F**) and the ankle-brachial index (in PAD stage II patients only). The r-squared and the *p*-values are shown within the graphs. Significant differences are highlighted in bold.

Plasma levels of pro- (i.e., VEGF) and anti- (i.e., TGFβ1) angiogenic factors also were examined in the study participants: VEGF levels were significantly elevated in PAD stage II patients compared to those in age- and sex-matched individuals without PAD diagnosis and a similar cardiovascular risk profile (*p* = 0.0198; Figure 4A). Plasma levels of active TGFβ1 did not significantly differ between both groups (*p* = 0.9586; Figure 4B) but were significantly higher in age-matched persons with risk factors compared to those without (*p* = 0.0454). In contrast to sEPCR, biomarkers of endothelial (dys-)function in plasma, that is, sE-selectin were not associated with plasma levels of VEGF (Figure 4C), whereas a significant association was observed for active TGFβ1 (Figure 4D). Neither VEGF nor active TGFβ1 plasma levels correlated with non-invasive readouts of the vascular status, that is, the ankle-brachial index (Figure 4E,F).

## 4. Discussion

Endothelial dysfunction is the earliest sign of vascular disease. It promotes the development of atherosclerotic lesions at predilection sites within the arterial tree, including the arteries of the lower (and less frequently, upper) extremities, resulting in peripheral artery disease. Whereas initially clinically silent (stage I), the obstruction of the arterial lumen results in the imbalance between perfusion and the oxygen and nutrient demands of the musculature, at first only during exercise (stage II) and later already at rest (stage III), and ischemic tissue damage and necrosis are the fatal outcome of this disease (stage IV). The diagnosis currently rests on clinical tests followed by invasive imaging procedures, and circulating biomarkers of endothelial dysfunction or inflammation may give additional information. These include inflammatory cell types, such as monocytes [31], or cytokines, such as TNFα and IL6, and acute-phase reactants like CRP, as well as markers of endothelial activation, such as VCAM1 and other adhesion molecules, reviewed in [32]. However, they are often unspecific and not used in clinical routine. Markers of defective endothelial angiogenic capacities may be of added value, and the first studies observed a significant reduction of endothelial progenitor cell numbers and function indices in PAD patients compared to healthy subjects [33,34].

We have recently shown that genetic deletion of EPCR in endothelial cells of genetically engineered mice results in impaired new vessel formation following experimental hindlimb ischemia [14]. In the present study, we tested the hypothesis that impaired angiogenesis in the lower extremities is associated with elevated circulating levels of EPCR, presumably shed from the endothelial cell surface, and that increased inflammation in those patients underlies the increased EPCR shedding. However, and in contrast to our initial hypothesis, we observed that sEPCR was reduced in patients with PAD stage II, and even further in those diagnosed with PAD stage III/IV. Our findings are in agreement with the presence of endothelial dysfunction in patients with PAD, and findings of elevated circulating levels of markers of endothelial cell function, that is, soluble E-selectin [35,36,37], further supported this conclusion. Importantly, plasma sEPCR levels, but not other biomarkers examined in our study (sE-selectin, sTie2, VEGF, or TGFβ), significantly correlated with the ankle-brachial index, strengthening a possible causal relationship between reduced endothelial EPCR expression and impaired vascular function. The presence of these associations should be taken into account when sEPCR levels are used as a biomarker. Our findings also corroborate previous reports of downregulated endothelial EPCR expression in patients with coronary atherosclerosis [38], or lower sEPCR levels in patients with acute ST-elevation myocardial infarction [10]. While our study was conceptualized to compare age- and sex-matched persons, we also examined a group of healthy individuals, 35 years of age or younger: The observed association of sEPCR with age and the age-associated increase in BMI should be considered when interpreting the data.

Endothelial activation and dysfunction are associated with increased shedding of receptors from the endothelial cell surface, including in patients with coronary artery disease [37]. However, plasma levels of ADAM17, a disintegrin and metalloproteinase 17 involved in the shedding of EPCR from the endothelial membrane [9], did not significantly differ between PAD stage II patients and age-matched healthy persons with no PAD diagnosis in our study. This finding may be related to the fact that immunoassays were used to determine protein levels and not a functional assay to assess the activity of this protease on endothelial cells. It is also possible that other proteases exist and are more relevant in the context of PAD, although this could not be examined in this study. ADAM17 (also known as TNFα-converting enzyme) has also been implicated in the proteolytic cleavage of TNFα from endothelial cells [39] or leucocytes [40], and the regulation of TNFα levels in inflammation [41]. However, like ADAM17, plasma levels of TNFα and IL1β did not differ between the groups. Of note, the detection of elevated serum NLRP3 and IL1β levels (using Multianalyte LegendPlex Flow Assay) in PAD patients undergoing limb amputation surgery suggested vascular inflammasome activation at this later stage [42]. Differences in the PAD disease severity, but also the techniques used to detect IL1β may underlie the discrepancy between the findings regarding circulating IL1β levels. In this regard, inflammatory mediators and markers of endothelial dysfunction and angiogenesis were not measured in PAD stage III/IV patients in our study. Targeting IL1β signaling, for example, by using antibodies (canakinumab), to treat PAD has been tested in a randomized controlled clinical trial and was found to improve the maximum and pain-free walking distance in patients with symptomatic PAD [43]. The therapeutic strategies recommended by the current guidelines for the management and treatment of PAD, including risk factor reduction, statins, or anti-platelet regimes, also may exert their protective effects by targeting inflammation, as recently reviewed in [44]. That the medication taken by PAD patients may have affected the findings of our study cannot be excluded.

In addition to alterations in EPCR shedding from an activated, dysfunctional endothelium, increased internalization and lysosomal degradation may have contributed to the observed changes in circulating EPCR levels, as reported for E-selectin [45]. Inflammation was also shown to alter the internalization and degradation of TNFα and thrombomodulin [46]. The presence of active inflammatory processes was suggested by findings of elevated white blood cell counts and hsCRP levels in PAD patients, and both parameters were associated with the serum glucose, but not plasma sEPCR levels or the ankle-brachial index. This observation is in line with larger clinical studies showing a role for inflammation accompanying the metabolic syndrome [47]. Soluble EPCR retains its binding affinity for protein C [48] and competes with membrane EPCR for protein C binding and protein C activation [49]. However, we did not observe significant differences in plasma protein C activity. Previous studies have shown that the cellular APC pathway exerts strong vasculoprotective effects, in addition to and independent of its anticoagulant properties, including anti-apoptotic and anti-inflammatory activities [50].

Interestingly, and despite the documented presence of reduced vascularization, plasma levels of VEGF, a major angiogenic factor, were elevated in patients with PAD and not associated with the ankle-brachial index. Local delivery of recombinant adenoviral VEGF did not improve intermittent claudication, walking time, or ankle-brachial index in patients with unilateral PAD [51]. Whether this points to defective or aberrant VEGF signaling in PAD has to be determined. In this regard, inflammation upregulates the expression of the protein tyrosine phosphatase-1B, a negative regulator of tyrosine kinase receptor signaling, on endothelial cells [52]. Plasma levels of soluble Tie2 receptors, involved in angiopoietin-1/2 signaling, did not differ between PAD patients and age-matched controls with no PAD diagnosis. Plasma levels of TGFβ1, a cytokine for which both pro- and anti-angiogenic functions have been reported, also did not differ in PAD patients, in contrast to a previous study, in which total, not active TGFβ1, levels were measured in patients with PAD [53].

The main limitation of the present study is the small number of age- and sex-matched patients examined in each group and that some of the data, in particular the ankle-brachial index, were available only from those with a clinical indication, and not from the healthy participants in the study. Measurements were also only performed at one point in time, and patients were not followed up later, precluding any conclusions regarding the potential implications of our findings for disease progression or prognosis. Also, only blood samples were available for analysis, limiting the possibilities for mechanistic studies to explain our results better.

## 5. Conclusions

The results of this pilot clinical study provide the first evidence of the potential role of reduced endothelial expression of EPCR, an anticoagulant with anti-inflammatory properties, as a biomarker of endothelial and vascular dysfunction in peripheral artery disease. Although differences in soluble EPCR levels in patients with peripheral ischemic disease and control subjects may have multifactorial causes, both related and unrelated to ischemia, future studies should examine in more detail the impact of preventive or therapeutic strategies targeting endothelial EPCR expression to modulate the angiogenic capacities of the endothelium.

## Figures and Tables

**Table 1:** Baseline characteristics of the study population.

	PAD Stage II	PAD Stage III/IV	Healthy Age-Matched with Risk Factors	Healthy Age-Matched	Healthy ≤35 Years	*p*-Values
Number	19	19	19	19	19	n.s.
Male sex (%)	9 (47.4%)	9 (47.4%)	9 (47.4%)	9 (47.4%)	9 (47.4%)	n.s.
Age, years	67.4 ± 8.1 ^####^	68.9 ± 10.8 ^####^	67.2 ± 7.3 ^####^	66.1 ± 9.5 ^####^	23.7 ± 4.0	****
BMI, kg/m^2^	27.6 ± 4.8 ^###^	26.8 ± 4.3 ^##^	26.7 ± 3.1 ^##^	25.7 ± 3.8 ^#^	21.9 ± 2.9	***
Diabetes mellitus (%)	6 (31.6%)	8 (42.1%)	2 (10.5%)	0	0	0.0878
Dyslipidemia (%)	14 (73.7%)	9 (47.3%)	6 (31.2%)	0	0	*
Hypertension (%)	16 (84.2%)	11 (57.9%)	14 (73.7%)	0	0	n.s.
Metabolic Syndrome (%)	8 (42.1%)	4 (21.1%)	4 (21.1%)	0	0	n.s.
Smoking, active (%)	7 (36.8%)	1 (5.2%) ^#^	1 (5.3%)	0	0	**
Smoking, former (%)	10 (52.6%)	13 (68.4%)	8 (42.1%)	10 (52.6%)	0	n.s.
Family history (%)	2 (10.5%)	4 (21.1%)	6 (31.2%)	2 (10.5%)	0	n.s.
ACE inhibitor/AT1 blocker (%)	12 (63.2%)	9 (47.4%)	13 (68.4%)	0	0	n.s.
Aspirin (%)	17 (89.5%)	11 (57.9%)	3 (15.8%)	0	0	****
β-blocker (%)	10 (52.6%)	6 (31.6%)	4 (21.1%)	0	0	n.s.
Calcium antagonists (%)	11 (57.9%)	3 (15.8%)	5 (26.3%)	0	0	*
DOACs/VKAs (%)	2 (10.5%)	5 (26.3%)	1 (5.3%)	0	0	n.s.
Fibrates (%)	1 (5.3%)	0	0	0	0	n.s.
Nitrates (%)	0	1 (5.3%)	0	0	0	n.s.
P2Y12 antagonists (%)	2 (10.5%)	8 (42.1%)	1 (5.3%)	0	0	**
Statins (%)	15 (78.9%)	9 (47.4%)	4 (21.1%)	0	0	**

Data are given as mean ± standard deviation or as absolute numbers (and percentages). * *p* < 0.05, ** *p* < 0.01, *** *p* < 0.001 and **** *p* < 0.0001, as determined using One-Way-ANOVA (or Kruskall–Wallis), and ^#^ *p* < 0.05, ^##^ *p* < 0.01, ^###^ *p* < 0.001 and ^####^ *p* < 0.0001 vs. healthy persons < 35 years of age, as determined using Tukey’s (or Dunn’s) multiple comparisons test. n.s., non-significant. Categorical parameters were compared in age-matched healthy persons with risk factors and patients with PAD stage II and stage III/IV using a Chi-Square test for (only PAD II vs. PAD III/IV). Abbreviations: ACE, angiotensin-converting enzyme; AT1, angiotensin II receptor type 1; BMI, body mass index; DOAC, direct-acting oral anticoagulant; PAD, peripheral artery disease; VKA, vitamin-K antagonist.

**Table 2:** Baseline laboratory parameters of the study population.

	PAD Stage II	PAD Stage III/IV	Healthy Age-Matched with Risk Factors	Healthy Age-Matched	Healthy ≤35 Years	*p*-Value
n	19	19	19	19	19	
Glucose, mg/dL	102 ± 14 ^&&^	134 ± 52 ^#### §§§§ &^	91 ± 6.2	88 ± 5.5	86 ± 12	**
Triglycerides, mg/dL	161 ± 70 ^§§^	120 ± 46	118 ± 62	87 ± 29	119 ± 60	**
Total cholesterol, mg/dL	193 ± 35 ^§ &&^	178 ± 54 ^§§§ &&&&^	242 ± 39 ^##^	234 ± 36 ^#^	194 ± 24	****
LDL cholesterol, mg/dL	101 ± 31 ^§§§ &&&&^	101 ± 44 ^§§§ &&&&^	153 ± 38 ^###^	151 ± 31 ^###^	105 ± 16	****
HDL cholesterol, mg/dL	59 ± 17	53 ± 17	65 ± 18	65 ± 13	65 ± 21	n.s.
C-reactive protein, mg/L	2.6 ± 1.6 ^#^	19 ± 21 ^#### §§§ &&&^	1.2 ± 0.7	1.4 ± 1.4	1.3 ± 1.4	****
Creatinine, mg/dL	0.87 ± 0.15	0.97 ± 0.37	0.87 ± 0.16	0.92 ± 0.18	0.87 ± 0.13	n.s.
White blood cell count, ×10^3^/μL	7.9 ± 1.4	11.7 ± 7.6 ^#### §§§ &&&& %^	6.0 ± 1.4	6.2 ± 1.9	5.9 ± 1.0	****
Red blood cell count, ×10^6^/μL	4.6 ± 0.6	4.3 ± 0.6 ^# §^	4.6 ± 0.4	4.7 ± 0.5	4.7 ± 0.3	*
Hematocrit %	43 ± 4.3	42 ± 13	43 ± 2.7	43 ± 4.4	42 ± 3.1	n.s.
Hemoglobin, g/dL	14.2 ± 1.3	14.1 ± 6.0	14 ± 1.0	14 ± 1.5	14 ± 1.3	n.s.
Platelets, ×10^3^/μL	263 ± 58	285 ± 114	264 ± 51	230 ± 33	245 ± 45	n.s.

Data are given as mean ± standard deviation or as absolute numbers (and percentages). * *p* < 0.05, ** *p* < 0.01, and **** *p* < 0.0001, as determined using One-Way-ANOVA (or Kruskall–Wallis for CRP), and ^#^ *p* < 0.05, ^##^ *p* < 0.01, ^###^ *p* < 0.001 and ^####^ *p* < 0.0001 vs. healthy persons < 35 years of age, ^§^ *p* < 0.05, ^§§^ *p* < 0.01, ^§§§^ *p* < 0.001 and ^§§§§^ *p* < 0.0001 vs. healthy, aged-matched persons without risk factors, ^&^ *p* < 0.05, ^&&^ *p* < 0.01, ^&&&^ *p* < 0.001 and ^&&&&^ *p* < 0.0001 vs. healthy, aged-matched persons with risk factors, and ^%^ *p* < 0.05 vs. PAD stage II, as determined using Tukeys (or Dunn’s) multiple comparisons test. n.s., non-significant. Categorical parameters were compared in age-matched healthy persons with risk factors and patients with PAD stage II and stage III/IV using a Chi-Square test for (only PAD II vs. PAD III/IV). Abbreviations: HbA1c, glycosylated hemoglobin; HDL, high-density lipoprotein; LDL, low-density lipoprotein; PAD, peripheral artery disease.

## Data Availability

Any personal or patient data are unavailable due to privacy or ethical restrictions. All other data are available from the corresponding author upon reasonable request.
